# p53 and PCNA Expression in Keratocystic Odontogenic Tumors Compared with Selected Odontogenic Cysts

**Published:** 2013

**Authors:** Maryam Seyedmajidi, Shima Nafarzadeh, Sepideh Siadati, Shahryar Shafaee, Ali Bijani, Nazanin Keshmiri

**Affiliations:** 1 *Dental Materials Research Center, Dental Faculty, Babol University of Medical Sciences, Babol, Iran.*; 2 *Oral and Maxillofacial Pathology Department, Dental Faculty, Babol University of Medical Sciences, Babol, Iran.*; 3 *Pathology Department, Medical Faculty, Babol University of Medical Sciences, Babol, Iran.*; 4 *Cellular and Molecular Biology Research Center (CMBRC), Babol University of Medical Sciences, Babol, Iran.*; 5 *Non-Communicable Pediatrics Diseases Research Center, Babol University of Medical Sciences, Babol, Iran.*; 6 *Student Research Committee, Babol University of Medical Sciences, Babol, Iran.*

**Keywords:** p53, PCNA, radicular cyst, dentigerous cyst, keratocystic odontogenic tumor, calcifying cystic odontogenic tumor

## Abstract

p53 and PCNA expression in keratocystic odontogenic tumors compared with selected odontogenic cysts Summary: The aim of this study was to evaluate p53 and PCNA expression in different odontogenic lesions regarding their different clinical behaviors. Slices prepared from 94 paraffin-embedded tissue blocks (25 radicular cysts (RC), 23 dentigerous cysts (DC), 23 keratocystic odontogenic tumors (KCOT) and 23 calcifying cystic odontogenic tumors (CCOT)) were stained with p53 and PCNA antibodies using immunohistochemistry procedure. The highest level of p53 expression was in the basal layer of RC, and the highest level of PCNA expression was in the suprabasal layer of KCOT. The differences of p53 expression in basal and suprabasal layers as well as PCNA expression in the suprabasal layer were significant but there was no significant difference in PCNA expression in the basal layer of these lesions. The expression of p53 in the basal layer of RC was higher than in other cysts. This may be due to intensive inflammatory infiltration. Also, the high level of PCNA expression in the suprabasal layer of KCOT may justify its neoplastic nature and tendency to recurrence. KCOT and calcifying cystic odontogenic tumors did not show similar expression of studied biomarkers.

Odontogenic cysts are common lesions in oral cavity and in spite of similar histo-pathological features have different clinical behaviors. In rare cases, squamous cell carcinoma in radicular cyst is reported. Also, epithelial layer of dentigerous cyst may transform to ameloblastoma, squamous cell carcinoma or intraosseous mucoepidermoid carcinoma. Odontogenic keratocyst (OKC) has specific histopathological and clinical features, different histopathogenesis and biological behavior. Many authors believe that dentigerous and radicular cysts enlarge because of an increase in intraluminal osmotic pressure. However, enlargement of OKC may be a result of unknown native epithelial factors or enzymatic activity of fibroconnective tissue in the cyst wall. In 2005, WHO workshop considered parakeratinized OKC as a cystic neoplasm and recommended the term Keratocystic Odontogenic Tumor (KCOT). Pathogenic mechanisms that cause growth and enlargement of KCOT includes high proliferation rate, high expression of antiapoptotic proteins like bcl-2 and expression of matrix metalloproteinases (MMPs) 2 and 9. It seems that KCOT has a neoplastic potential. Epithelial dysplasia and squamous cell carcinoma may rarely occur, but there are different findings about ameloblastomatous changes (1-2).

Calcifying odontogenic cyst (COC) has a variety of histopathological and clinical behaviors. Most of the time, it seems like a non-neoplastic cyst, whereas in other times it has no cystic property which may be invasive or even malignant and is therefore considered as a neoplasm. In some conditions, it may also be seen withother odontogenic tumors including odontoma, adenomatoid odontogenic tumor and amelo-blastoma. In WHO classification, COC and all its subtypes are considered as odontogenic tumor (1-2).

p53 protein is a product of Tp53 (Tumor protein p53), a tumor suppressor gene that is expressed in G1 phase of the cell cycle. It helps DNA damage repairs and prevents cells to enter S phase. If DNA is repaired, cell cycle arrest is ended. But if DNA is not successfully repaired, p53 induces apoptosis and leads the cell to die. In the absence or mutations of p53, DNA damage can not be repaired and it leads to proliferation of damaged cells and malignant transformation (3-4).

In normal non - stressed cells, p53 has a short half-life of about 20 minutes. As a result of cellular stress (e.g.DNA assault and damage), p53 undergoes posttranscriptional modifications. It is protected from the effects of MDM2 (Murine Double Minute 2) and its half-life increases (5-9).

The cellular proliferation increase has an important role in the progression of odontogenic cysts. p53 protein has an important role in controlling the expression of cellular proliferation inhibiting genes. Mutation in p53 gene can prevent its inhibitory role, resulting in oncogenic activities and neoplastic changes. Wild type p53 protein is expressed in small amounts, has a short half-life and cannot be detected using immunohistochemical procedure. This detection can be possible in conditions where the protein is expressed in large amounts or is accumulated in mutant cells (10-11).

Overexpression of p53 in lesions without mutation in the corresponding gene or even in normal tissues was reported by Cruz et al. and Pillai et al. In these tissues, positive results are due to the persistence of wild type p53 protein by some unknown reasons and consequently its concen-tration increase leading to the possibility of its detection by immunohistochemistry procedure (12-13).

PCNA (Proliferating Cell Nuclear Antigen), is a nuclear protein essential for nucleic acid metabolism in DNA transcription and repair. It is expressed in high amount in growing cells during cell cycle. The expression of PCNA can be used as a cell proliferative marker, because proliferating cells remain for a longer time in G1/S phase (14). Increased expression of PCNA may be stimulated by growth factors or as a result of DNA injury in the absence of cell cycle (15).

Considering the roles and effects of p53 and PCNA in cells proliferation, this study was performed to understand the behavior of epithelial cells in different odontogenic cysts (radicular cyst as an inflammatory cyst, dentigerous cyst as a developmental cyst, calcifying cystic odontogenic tumor and KCOT which are tumoral lesions with cystic appearance) regarding their clinical behavior differences, tendency to neoplastic transformation and recurrence after treatment.

## Materials and Methods

After the approval of the Institutional Ethics Committee, 94 paraffin - embedded tissue blocks including 25 radicular cysts, 23 dentigerous cysts, 23 KCOTs and 23 calcifying cystic odontogenic tumors collected between March 2003 and February 2010, were obtained from the archives of Oral & Maxillofacial Pathology Department of Babol University of Medical Sciences, Babol, Iran.

Tissue blocks were cut into 5 microns slices, stained with hematoxylin & eosin and reviewed by Oral & Maxillofacial pathologist. Blocks containing maximum epithelial length upon gross review were selected and were cut into 3 microns slices. Tissue slides were deparaffinized in xylene and rehydrated through graded concentrations of ethanol (absolute ethanol, 96% ethanol, 80% ethanol and 70% ethanol).

After washing with tap water, the slides were placed in sodium citrate buffer (PH=6.0) at 120ºC, for 12 min. The slides were chilled at room temperature for 15 min, rinsed in tap water and placed in Tris Buffered Saline (TBS) for 5 min.

The slides were incubated in hydrogen peroxide for 10 min, then rinsed first in tap water then in TBS for 5 min.

The slides were covered by monoclonal antibody for p53 (Clone D07, Isotype; IgG2b Kappa, DakoCytomation, Glostrup, Denmark) and PCNA (Clone PC10, Isotype; IgG2a Kappa, DakoCytomation, Glostrup, Denmark) for 1 h, then rinsed in tap water and placed in TBS for 5 min.

After immunostaining, the slides were coun-terstained with Meyer's hematoxylin, mounted with entellan and coverslide and examined by light microscopy (Olympus BX41, Shibuya-Ku, Tokyo, Japan) under 400X magnification.

Breast carcinoma was used as the positive control and omission of the primary antibody was the negative control (11).

The brown stained nuclei of epithelial cells were considered as positive. The percentages of positive cells in the epithelium of cysts in basal and suprabasal layers (100 cells in 10 high power fields) were evaluated by two pathologists. The review of microscopic slides was performed by using a trinocular optic microscope and interobserver agreement was seen in all cases.

A score index of 1, 2, 3, 4 and 5 corresponding to staining in < 1%, 1-10%, 11-33%, 34-66% and >67% of epithelial cells, was used respectively (16).

The data were analyzed using Statistical Package for the Social Sciences (version 17), Kruskal Wallis test for comparing the mean percentage of positive cells in four groups and Wilcoxon Signed Ranks test for comparing the mean percentage of positive cells in basal and suprabasal layers between four groups. P<0.05 was considered as significant.

## Results

**Fig 1:**
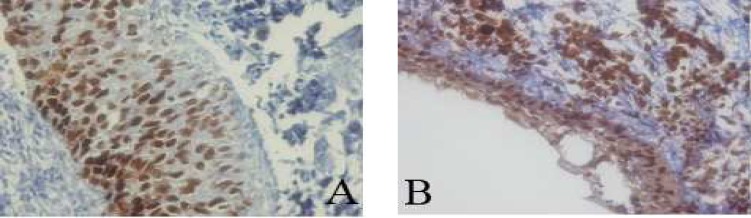
Immunohistochemical expression of p53 (A) and PCNA (B) in radicular cyst (400X).

**Fig 2:**
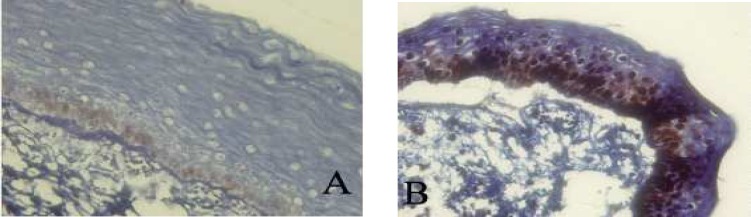
Immunohistochemical expression of p53 (A) and PCNA (B) in keratocystic odontogenic tumor (400X).

**Fig 3:**
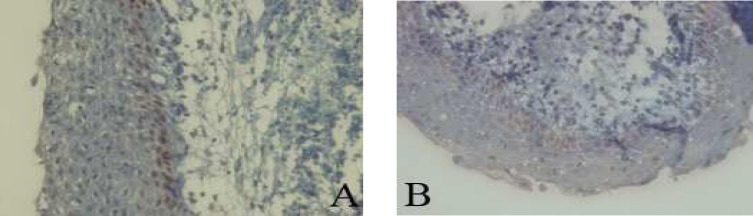
Immunohistochemical expression of p53 (A) and PCNA (B) in calcifying cystic odontogenic tumor (400X).

**Fig 4:**
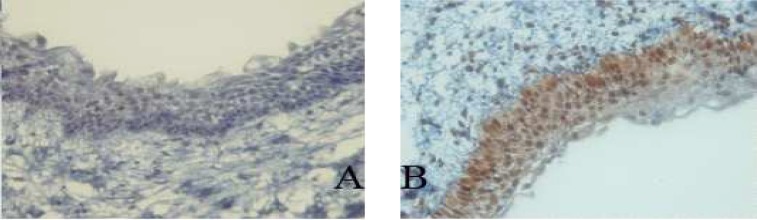
Immunohistochemical expression of p53 (A) and PCNA (B) in dentigerous cyst (400X) (400X).

The percentage of p53 and PCNA stained cells in basal and suprabasal layers of radicular cyst, dentigerous cyst, KCOT and calcifying cystic odontogenic tumor (CCOT) are shown in Table 1. Figures 1-4 are the representatives of immuno-histochemical stainings.

**Chart 1:**
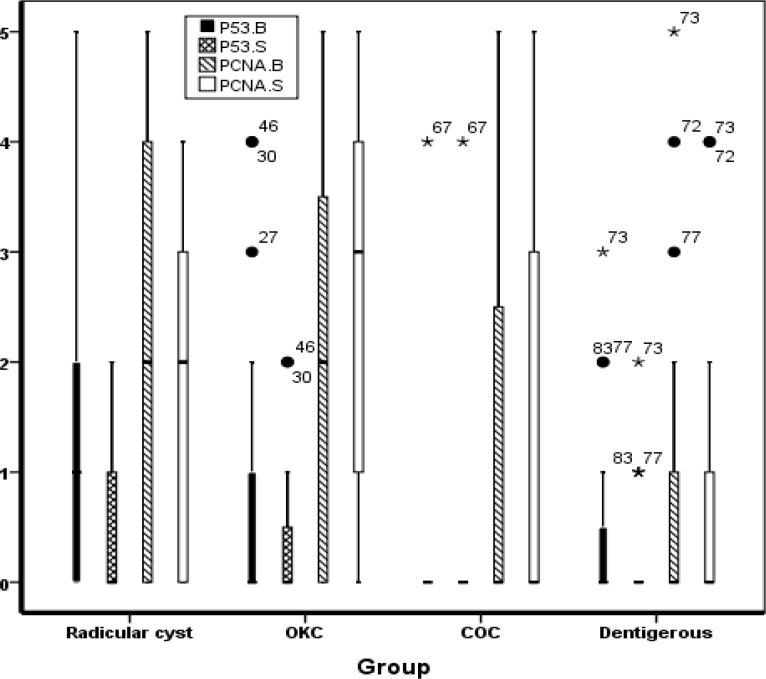
p53 and PCNA expression in basal and suprabasal layers in different odontogenic cysts

The highest level of p53 expression was in the basal layer of radicular cyst followed by KCOT and dentigerous cyst and the least amount belonged to CCOT. The same order was true in suprabasal layers. The highest level of PCNA expression was seen in suprabasal layers of KCOT, followed by radicular cyst, CCOT and dentigerous cyst. This result was also correct for the basal layer (Chart 1).

The percentage of p53 and PCNA stained cells in the basal and suprabasal layers of four types of cysts were compared using Kruskal-Wallis test. The differences between PCNA expression in the basal layer of these cysts were not significant (P=0.090) but the differences between the expression of P53 in both basal and suprabasal layers of these cysts as well as the differences between the expression of PCNA in the suprabasal layer of these cysts were significant (P=0.008, P=0.031 and P= 0.009, respectively). 

The percentages of stained cells for p53 and PCNA were compared in the basal and suprabasal layers of each cyst, using Wilcoxon Signed Ranks test. P53 expression in the basal layer of radicular 

**Table 1:** The expression of p53 and PCNA in odontogenic cysts based on percentage of stained cells in basal and suprabasal layers cyst, dentigerous cyst and KCOT was higher than in the suprabasal layer (P=0.007, 0.024 and 0. 025, respectively), but there was no significant difference in CCOT (P=1.000).

Site of expression of marker	Score 5	Score4	Score3	Score2	Score1	unstained	Type of Marker	Numbers of cyst	Type of cyst
Basal layer	4%(1)	0%(0)	8%(2)	20%(5)	20%(5)	48%(12)	P53	25	Radicular cyst
Suprabasal layer	0%(0)	0%(0)	0%(0)	20%(5)	20%(5)	60%(15)			
Basal layer	20%(5)	8%(2)	16%(4)	8%(2)	4%(1)	44%(11)	PCNA		
Suprabasal layer	0%(0)	12%(3)	24%(6)	16%(4)	0%(0)	48%(12)			
Basal layer	0%(0)	8.7%(2)	4.3%(1)	13%(3)	0%(0)	73.9%(17)	P53	23	Keratocystic odontogenic tumor
Suprabasal layer	0%(0)	0%(0)	0%(0)	8.7%(2)	17.4%(4)	73.9%(17)			
Basal layer	21.7%(5)	4.3%(1)	13%(3)	26.1%(6)	0%(0)	34.8%(8)	PCNA		
Suprabasal layer	13%(3)	21.7%(5)	21.7%(5)	17.4%(4)	0%(0)	26.1%(6)			
Basal layer	0%(0)	4.3%(1)	0%(0)	0%(0)	0%(0)	95.7%(22)	P53	23	Calcifying Cystic odontogenic tumor
Suprabasal layer	0%(0)	4.3%(1)	0%(0)	0%(0)	0%(0)	95.7%(22)			
Basal layer	8.7%(2)	8.7%(2)	8.7%(2)	17.4%(4)	4.3%(1)	52.2%(12)	PCNA		
Suprabasal layer	4.3%(1)	13%(3)	13%(3)	17.4%(4)	0%(0)	52.2%(12)			
Basal layer	0%(0)	0%(0)	4.3%(1)	8.7%(2)	13%(3)	73.9%(17)	P53	23	Dentigerous cyst
Suprabasal layer	0%(0)	0%(0)	0%(0)	4.3%(1)	13%(3)	82.6%(19)			
Basal layer	4.3%(1)	4.3%(1)	4.3%(1)	4.3%(1)	21.7%(5)	60.9%(14)	PCNA		
Suprabasal layer	0%(0)	8.7%(2)	0%(0)	4.3%(1)	26.1%(6)	60.9%(14)			

In radicular cyst, PCNA expression in the basal layer was higher than in the suprabasal layer (P=0.003), but there was no significant difference in other cysts (KCOT, CCOT and dentigerous cyst, P=0.188, P=0.705 and P= 0.083, respectively). Therefore, the highest level of p53 and PCNA expression was found in the basal layer of radicular cyst and the suprabasal layer of KCOT, respectively.

## Discussion

We studied the expression of p53 and PCNA in some odontogenic cysts. p53 expression was higher in the basal layer of radicular cyst, followed by KCOT. PCNA expression was higher in the suprabasal layer of KCOT, followed by radicular cyst.

p53 expression in basal and suprabasal layers and PCNA expression in suprabasal layers between these cysts were significantly different but this was not true for PCNA expression in the basal layer of these cysts.

The results of this study showed that radicular cyst and KCOT had a high percentage of p53 positive cells in their basal layer. In KCOT, the suprabasal layer showed the highest expression of PCNA. These unique findings in KCOT pointed that proliferative components in this lesion are in basal and suprabasal layers. This finding may justify the neoplastic nature of KCOT and it may also explain the clinical behavior and tendency to recurrence of this lesion. There are some studies with different results that evaluated the expression of p53 and PCNA for understanding epithelial cells behavior in different odontogenic cysts.

According to Mighell et al., the defferent results are due to complex biology of p53 and PCNA, histological preparation and immuno-histochemical grading protocol used. Although, the type of lesion, etiology and clinical behavior of lesions should be taken into consideration (11).

Carvalhais et al. studied p53 expression in odontogenic cysts and tumors. They did not find any positive cells (17).

The relationship between p53 expression and cell proliferation was showen in de Oliveira et al.'s study. They concluded that p53 expression was seen in proliferating cells, but it accumulated due to several factors such as cellular stress. Also, they showed that in radicular and dentigerous cysts, the expression of p53 and PCNA is a response to cellular stress resulting from inflammation, even in the cases of developmental cyst such as dentige-rous cyst (11).

Studies showed that growth factors and cytokines (Interleukin 1, Interleukin 6 and tumor necrosis factor) are released in inflammatory processes. Inflammation may cause cell pro-liferation and inflammatory cytokines may produce cellular stress (18).

In dentigerous cyst, the inflammatory stimulus may be the result of the eruption process that caused cellular proliferation which might be present for a short time (11). This could explain the lower expression of p53 and PCNA in dentigerous cyst. It was not proven that inflammatory stimuli could cause dentigerous cyst, but usually in the connective tissue of cyst, there was an inflammatory infiltration that might have caused epithelial cells proliferation.

In radicular cyst, the inflammatory stimuli are a result of persistent bacterial contaminations of the root canal. This has an effect on epithelial cells, especially in the basal layer and produces the increase and maintenance of the proliferative activity (11).

Therefore, the high expression of p53 and PCNA in radicular cysts is a reflection of the cellular stress and proliferation induced by inflammation that can inhibit the degradation of p53 and increase the level of PCNA (11).

The relationship between inflammation and cell proliferation in KCOT was studied by de Paula et al. They used AgNOR staining, Ki67 and PCNA immunohistochemistry for their analysis and concluded that the expression of biomarkers in this lesion showed a cellular proliferation pattern consistent with neoplastic cells and independent of inflammation. The high levels of p53 and PCNA expression in the suprabasal layer, can explain why KCOT has a proliferation and maturation pattern that differs from other lesions. These findings may explain its different clinical behaviors and the tendency of recurrence (19).

Kaplan and Hirschberg studied the areas with and without inflammation in KCOT and did not find a significant difference in their proliferation rate. They concluded that inflammation in KCOT did not have any effect on its proliferative potential (20). Also, in the present study all of KCOTs were non-inflamed.

Gallana-Alvarez and Wagner showed that the neoplastic cells in calcifying odontogenic cyst may have an increasing proliferative potential. Calcifying odontogenic cyst is considered as a tumoral lesion and the presence of mutant proteins should be taken into consideration. P53 expression may be related to the proliferation rate in this lesion (21-22).

However, in the present study, there was no high expression of p53 and PCNA in CCOT in comparison to other cysts. This differs from the study of de Oliveira who found a high level of p53 and PCNA in calcifying odontogenic cysts (11).

In our study, a significant difference between the percentages of stained cells was found for both markers in basal and suprabasal layers of radicular cyst, with more stained cells in basal layer, demonstrating that in radicular cyst, basal layer is the proliferative component. In KCOT and dentigerous cyst, a difference between basal and suprabasal layers was only seen for e P53 expression and no significant difference for PCNA expression was found, indicating a similar expression of PCNA in both basal and suprabasal layers of dentigerous cyst and KCOT. This can be related to the similar proliferative potential of the two layers in these lesions.

The study of Wagner was performed on radicular cyst, dentigerous cyst and KCOT. p53 was just expressed in KCOT indicating different clinical behaviors of this cyst (23).

In the present study, the high expression of PCNA in the suprabasal layer of KCOT in comparison to other cysts indicates that the suprabasal layer of this cyst is a proliferative component and explains the different growth patterns. Also, the high expression of p53 in basal layer of radicular cyst indicates that inflammatory stimuli produced from persistent bacterial contamination of root canal could induce cellular stress (11).

In KCOT, PCNA expression may show a proliferative pattern similar to neoplastic cells.

On the other hand, the presence of mutant p53 in KCOT must be taken in consideration. This was explained by Gonzales-Moles using antibodies specific to mutant p53 (24).

In KCOT, the epithelium has a little intrinsic growth potential that is not seen in epithelial cells of other cysts (1). Li et al. demonstrated that the epithelium of KCOT has a suprabasal proliferative component (15). The number of positive cells for PCNA in the epithelium of KCOT were significantly higher than dentigerous and radicular cysts. This finding was described by Li et al. and Piattelli et al. (15, 25).

p53 expression in the basal layer of radicular cyst was more than other cysts. It might be a result of inflammation. Also, the high expression of PCNA protein in the suprabasal layer of KCOT might be a result of clinical behavior and its tendency to recurrence. On the other hand, this lesion showed a proliferation pattern different from those found in other lesions (11). Two tumoral lesions, KCOT and CCOT were not similar in p53 and PCNA expression.

## Conflict of interest

Authors declared no conflict of interest.

## References

[1] Damm DD, Bouquot JE, Neville BW (2009). Oral and Maxillofacial Pathology.

[2] Regezi JA, Sciubba JJ, Jordan RCK (2011). Oral pathology. clinical pathology correlations.

[3] Levine AJ (1997). the cellular gatekeeper for growth and division. Cell.

[4] Nylander K, Dabelsteen E, Hall PA ( 2000). The p53 molecule and its prognostic role in squamous cell carcinomas of the head and neck. J Oral Pathol Med.

[5] Olson MO (2004). Sensing cellular stress: another new function for the nucleolus?. Sci STKE.

[6] Olson MO, Dundr M (2005). The moving parts of the nucleolus. Histochem Cell Biol.

[7] Stricker TP, Kumar V, Kumar V, Abbas AK, Fausto N (2010). Neoplasia. Robbins and Cotran pathologic basis of disease.

[8] Agarwala SS (1996). Paraneoplastic syndromes. Med Clin North Am.

[9] Chang F, Syrjanen S, Syrjanen K ( 1995). Implications of the p53 tumor-suppressor gene in clinical oncology. J Clin Oncol.

[10] Ozveren A, Tuskan C, Yaltirik M (2003). Expression or the tumor suppressor gene p53 in odontogenic cyst. Turk J Med Sci.

[11] de Oliveira MG, Lauxen Ida S, Chaves AC ( 2008). Immunohistochemical analysis of the patterns of p53 and PCNA expression in odontogenic cystic lesions. Med Oral Patol Oral Cir Bucal.

[12] Cruz IB, Snijders PJ, Meijer CJ (1998). p53 expression above the basal cell layer in oral mucosa is an early event of malignant transformation and has predictive value for developing oral squamous cell carcinoma. J Pathol.

[13] Pillai G, Roberts H, Gatter K ( 2003). p53 expression in normal paraffin-embedded tissue using different antibodies and antigen retrieval buffer systems. Histopathology.

[14] Kelman Z (1997). PCNA: structure, functions and interactions. Oncogene.

[15] Li TJ, Browne RM, Matthews JB (1994). Quantification of PCNA+ cells within odontogenic jaw cyst epithelium. J Oral Pathol Med.

[16] Allred DC, Harvey JM, Berardo M ( 1998). Prognostic and predictive factors in breast cancer by immunohistochemical analysis. Mod Pathol.

[17] Carvalhais J, Aguiar M, Araujo V ( 1999). p53 and MDM2 expression in odontogenic cysts and tumours. Oral Dis.

[18] Hudson JD, Shoaibi MA, Maestro R (1999). A proinflammatory cytokine inhibits p53 tumor suppressor activity. J Exp Med.

[19] de Paula AM, Carvalhais JN, Domingues MG ( 2000). Cell proliferation markers in the odontogenic keratocyst: effect of inflammation. J Oral Pathol Med.

[20] Kaplan I, Hirshberg A (2004). The correlation between epithelial cell proliferation and inflammationin odontogenic keratocyst. Oral Oncol.

[21] Reyes D, Villanueva J, Espinosa S ( 2007). Odontogenic calcificant cystic tumor: a report of two clinical cases. Med Oral Patol Oral Cir Bucal.

[22] Gallana-Alvarez S, Mayorga-Jimenez F, Torres-Gomez FJ (2005). Calcifying odontogenic cyst associated with complex odontoma: case report and review of the literature. Med Oral Patol Oral Cir Bucal.

[23] Wagner Y, Filippi A, Kirschner H (1999). [Cytokeratin and p53 expression of odontogenic cysts]. Mund Kiefer Gesichtschir.

[24] Gonzalez-Moles MA, Mosqueda-Taylor A, Delgado-Rodriguez M (2006). Analysis of p53 protein by PAb240, Ki-67 expression and human papillomavirus DNA detection in different types of odontogenic keratocyst. Anticancer Res.

[25] Piattelli A, Fioroni M, Santinelli A ( 2001). P53 protein expression in odontogenic cysts. J Endod.

